# A Systematic Review of Hepatitis E Virus Detection in Camels

**DOI:** 10.3390/vetsci10050323

**Published:** 2023-04-28

**Authors:** Sérgio Santos-Silva, Mahima Hemnani, Pedro Lopez-Lopez, Helena M. R. Gonçalves, António Rivero-Juarez, Wim H. M. Van der Poel, Maria São José Nascimento, João R. Mesquita

**Affiliations:** 1 ICBAS—Instituto de Ciências Biomédicas Abel Salazar, Universidade do Porto, 4050-313 Porto, Portugal; up202110051@edu.icbas.up.pt (S.S.-S.); up202110040@edu.icbas.up.pt (M.H.); 2 Grupo de Virología Clínica y Zoonosis, Unidad de Enfermedades Infecciosas, Instituto Maimónides de Investigación Biomédica de Córdoba, Hospital Reina Sofía, Universidad de Córdoba, 14004 Córdoba, Spain; lopezlopezpedro07@gmail.com (P.L.-L.); arjvet@gmail.com (A.R.-J.); 3 CIBER de Enfermedades Infecciosas (CIBERINFEC) Instituto de Salud Carlos III, 28220 Madrid, Spain; 4 Biosensor Ntech-Nanotechnology Services, Lda, Avenida da Liberdade, 249, 1° Andar, 1250-143 Lisboa, Portugal; helenardrgs@gmail.com; 5 REQUIMTE, Instituto Superior de Engenharia do Porto, 4200-072 Porto, Portugal; 6 Quantitative Veterinary Epidemiology Group, Wageningen University, 6708 PB Wageningen, The Netherlands; wim.vanderpoel@wur.nl; 7 Department Virology & Molecular Biology, Wageningen Bioveterinary Research, 8200 AB Lelystad, The Netherlands; 8 Faculdade de Farmácia, Universidade do Porto (FFUP), 4050-313 Porto, Portugal; saojose@ff.up.pt; 9 Epidemiology Research Unit (EPIUnit), Instituto de Saúde Pública da Universidade do Porto, 4050-600 Porto, Portugal; 10 Laboratório Para a Investigação Integrativa e Translacional em Saúde Populacional (ITR), 4050-600 Porto, Portugal

**Keywords:** hepatitis E virus, camel, zoonotic, infection

## Abstract

**Simple Summary:**

Acute hepatitis, which is a rising public health issue globally, is mostly caused by the hepatitis E virus (HEV). There is a potential risk of camel-borne zoonotic HEV infection in the desert regions of the Middle East and Africa, where camels frequently interact with human populations and camel-derived food products constitute a component of the food chain. To better understand the current state of this subject, the current work’s objective is to provide a scientific review of the detection of HEV genotypes seven and eight in camels around the world. Until today, no review paper has been published compiling and discussing the reports available on HEV in camels. More studies are required to ascertain the prevalence of HEV infection in camels worldwide. Additionally, because camels are utilized as a form of transportation in many countries and because HEV in these animals may pose a threat to public health, there is a possibility of foodborne transmission through contaminated camel products.

**Abstract:**

Hepatitis E virus (HEV) represents a major cause of acute hepatitis and is considered an emerging public health problem around the world. In the Middle East’s and Africa’s arid regions, where camels frequently interact with human populations and camel-derived food products are a component of the food chain, camel-borne zoonotic HEV infection is a potential threat. To date, no review paper has been published on HEV in camels. As such, the purpose of the current work is to provide a scientific review of the identification of HEV genotypes seven and eight in camels worldwide to have a better understanding of the current status of this topic and to identify gaps in the current knowledge. Searches were carried out in the electronic databases PubMed, Mendeley, Web of Science, and Scopus, including studies published until 31 December 2022 (*n* = 435). Once the databases were checked for duplicate papers (*n* = 307), the exclusion criteria were applied to remove any research that was not relevant (*n* = 118). As a result, only 10 papers were found to be eligible for the study. Additionally, in eight of the ten studies, the rates of HEV infection were found to be between 0.6% and 2.2% in both stool and serum samples. Furthermore, four studies detected HEV genotype seven in dromedary camels, and two studies have shown HEV genotype eight in Bactrian camels. Interestingly, these genotypes were recently reported in camels from the Middle East and China, where one human infection with HEV genotype seven has been associated with the consumption of contaminated camel meat and milk. In conclusion, more research will be needed to determine the prevalence of HEV infection in camels around the world as well as the risk of foodborne transmission of contaminated camel products. As camels are utility animals in several countries, HEV in these animals may pose a potential risk to public health.

## 1. Introduction

Hepatitis E virus (HEV) is a leading cause of acute hepatitis globally [1], which is an increasing concern for public health worldwide as it is an emerging issue [2]. In 2022, the International Committee on the Taxonomy of Viruses (ICTV) updated its classification of viruses and categorized HEV as a member of the *Hepeviridae* family. This family is further divided into two subfamilies, namely *Parahepevirinae* and *Orthohepevirinae* [3]. The *Parahepevirinae* subfamily infects trout and salmon, whereas the *Orthohepevirinae* subfamily infects mammals and birds and is further divided into four genera: *Paslahepevirus*, *Avihepevirus*, *Rocahepevirus*, and *Chirohepevirus*. HEV is a small virus that is quasi-enveloped with an icosahedral capsid that encloses the viral genome [4]. The genome is a single-stranded RNA molecule that is positive-sense and ranges from 6.4 to 7.3 kb in length, containing four partially overlapping open reading frames (ORF1, ORF2, ORF3, and ORF4) [5,6]. 

The *Paslahepevirus* genus consists of two species: *P. balayani*, which includes HEV genotypes capable of infecting humans and various mammalian species [7], and *P. alci*, which infects moose. Through a complete genome analysis, researchers have identified eight separate genotypes within the *P. balayani* species [8], with four genotypes (one–four) responsible for human disease. Genotypes one and two cause acute or fulminant hepatitis E infections, which are limited to humans and often result in large epidemics in developing countries due to inadequate sanitation and lack of clean drinking water [9,10]. The main route of transmission of HEV-1 and HEV-2 is the fecal-oral route; furthermore, it has been observed to be transmitted via blood transfusion [11]. Moreover, HEV-3 and -4 infect numerous mammal host species including humans [3], with swine being the main host, but are also found in deer, ruminants, rabbits, dolphins, raccoons, lynxes, cats, dogs, and equids [12]. Infections by these genotypes usually present as asymptomatic and self-limiting [13]. However, it has been observed that there are some risk groups, such as immunocompromised people, pregnant women, and individuals with preexisting chronic liver disease, that may experience a more severe course of infection and have a poorer prognosis [5,14,15]. In addition, it has been observed that primarily HEV genotype three may present extrahepatic manifestations which are mainly neurological [16,17]. Moreover, HEV genotype three is mainly transmitted from animals to humans through the consumption of raw or undercooked pork meat, as well as pork and game meat products [18]. Additionally, it has been suspected through the consumption of shellfish [19]. Other routes of HEV transmission not related to the consumption of animal products are blood product transfusions and solid organ transplantation [20,21]. HEV genotypes five and six have only been identified in wild boars found in Japan [22]. Meanwhile, HEV genotypes seven and eight were recently discovered in camels from China and the Middle East [7,23,24,25,26,27], with only one human infection with HEV genotype seven reported to be due to consumption of contaminated camel meat and milk [28]. The phylogenetic analysis in this report demonstrated high homology between sequences identified in samples obtained from the patient with those identified in dromedary meat and milk [28]. 

There are two species of camels in the world: the dromedary (*Camelus dromedarius*), which has one hump, and the Bactrian (*Camelus bactrianus*), which has two humps [29]. Camels have a unique physiological characteristic, such as the presence of fatty humps that act as a food reserve and store energy [30], providing energy during drought conditions when food is scarce, and helping them to live in arid, semi-arid, mountainous, and especially desert areas [31]. Dromedary and Bactrian camel species occupy disparate geographic areas, with the dromedary primarily being found in Arabian deserts, Afghanistan, Iran, Central and South Asia [32], and in the Middle East, in the northern regions of Africa, some parts of Asia, and the Indian sub-continent. On the other hand, the Bactrian camel is only found in inner, Central, and East Asia. This includes countries such as China, Mongolia, Kazakhstan, Kyrgyzstan, Turkmenistan, Afghanistan, northern parts of Iran, India, Pakistan, and extending to eastern Turkey [30,33]. In many countries, camels have multiple and important social and economic roles [34]. Camels’ milk production and meat are perhaps the most important ones; they are frequently used to transport surplus milk to market hubs for sale and, in a similar manner, camels are used to transport drinking water from far-off watering wells for both humans and young livestock [34]. They are almost mainly used by family households as beasts of burden to transport their temporary homes and other goods when moving about the grazing pastures [34]. As households move, camels are also used to transport the young, the sick, the elderly, and young animals. Furthermore, camels hold a significant role in traditional social relations and status [34].

Today, it is recognized that camels can transmit several infectious diseases, including bluetongue, West Nile disease, African horse sickness, Rift Valley fever, peste des petits ruminants, and Middle East respiratory syndrome [35]. The influenza D virus, which primarily infects cattle, sheep, goats, and other livestock animals, was found with seroprevalence in camels in Kenya in 2017 (99% after seroprevalence) [36]. Furthermore, the influenza C virus has also been found in dromedary camels in Kenya [37]. 

HEV genotype seven, first found in dromedary camels of the United Arab Emirates [23] and classified as DcHEV, is usually found in this species. Genotype eight is described in Bactrian camels and it was first discovered in China [24], increasing the range of mammalian hosts for HEV. Camel-borne zoonotic HEV infection is typical of concern, especially in the dry areas of the Middle East and Africa, where HEV infection is common [38], and where camels are often in contact with human populations and camel-derived food products are part of the food chain [29]. To date, no review paper has been published compiling and discussing the reports on HEV in camels. Hence, the aim of the present work is to provide a scientific review on the identification of HEV genotypes seven and eight in camels worldwide in order to have a better understanding of the current status of this topic and to identify gaps in current knowledge.

## 2. Materials and Methods

### 2.1. Selection of Articles

Comprehensive research of articles was conducted using the electronic searches Mendeley, PubMed, Scopus, and Web of Science databases, taking into consideration studies that were published up until 31 December 2022. The systematic review followed the Preferred Reporting Items for Systematic Reviews and Meta-Analysis (PRISMA) criteria [39], and only studies that were published, indexed, and peer-reviewed were considered for inclusion. This review took into account language limitations, and only documents written in the English language were taken into consideration.

The search for relevant literature involved using specific keywords such as “HEV” or “Hepatitis E Virus” in combination with “camel” and was conducted on databases including Mendeley, PubMed, Scopus, and Web of Science. Papers that did not focus on the detection of HEV in camels or were not relevant to this review were excluded after reading their titles and abstracts. The full paper was read to clarify any information that was unclear in the title and abstract. 

Two independent researchers (S.S.-S. and M.H.) conducted the screening of the databases and extracted relevant information. Any disagreements between the investigators were resolved with discussion or by involving a third investigator. 

**Figure 1:**
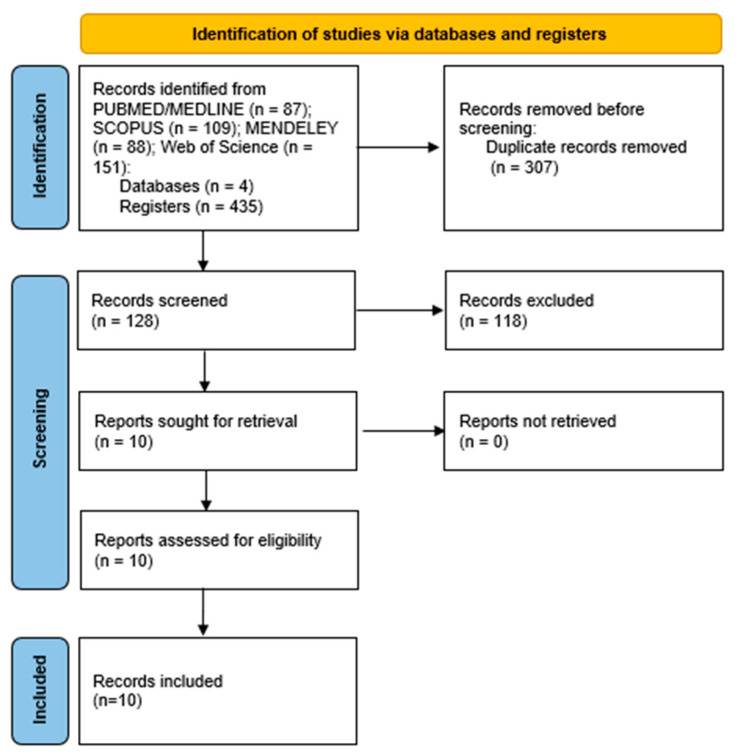
Flowchart following the Preferred Reporting Items for Systematic Reviews (PRISMA) guidelines.

Ten papers were identified as potentially suitable for the systematic review after applying the inclusion and exclusion criteria, and all of them were found to be eligible after being fully read. The selection process is illustrated in Figure 1.

### 2.2. Phylogenetic Analysis

The HEV sequences obtained from camels and present in GenBank database as of 9 February 2023 were analyzed phylogenetically. A phylogenetic tree was then constructed using the camel HEV sequences and reference sequences from HEV genotypes 1–6, based on the proposed reference sequences for subtypes of *Paslahepevirus balayani* [8], which were obtained from the NCBI (GenBank) nucleotide database (http://blast.ncbi.nlm.nih.gov/Blast, accessed on 9 February 2023). The camel HEV sequences were aligned and compared using MEGA version X software [40], while the Interactive Tree Of Life (iTOL) platform [41] was used to perform the phylogenetic analysis. In addition to the camel sequences present in GenBank, other representative sequences retrieved from the database were used for the analysis.

To carry out the analysis, the maximum-likelihood (ML) approach was utilized [40,42], as well as the General Time Reversible method to estimate the ML bootstrap values, which involved running 1000 replicates [42]. MEGA version X [40] determined that this model was the most suitable replacement model.

The calculation of base substitutions per site between sequences was performed, and the Tamura 3-parameter model [42] was used for analysis. A gamma distribution was used to model the rate variation among sites, with a shape parameter of 1. The analysis included codon positions 1st + 2nd + 3rd + Noncoding, and all uncertain positions were removed for each pair of sequences using the pairwise deletion option. The MEGA version X was utilized for carrying out evolutionary analyses [40].

## 3. Results

Table 1 presents a summary of the findings and conclusions of the 10 papers that were obtained from the electronic search conducted on the four databases.

**Figure 2:**
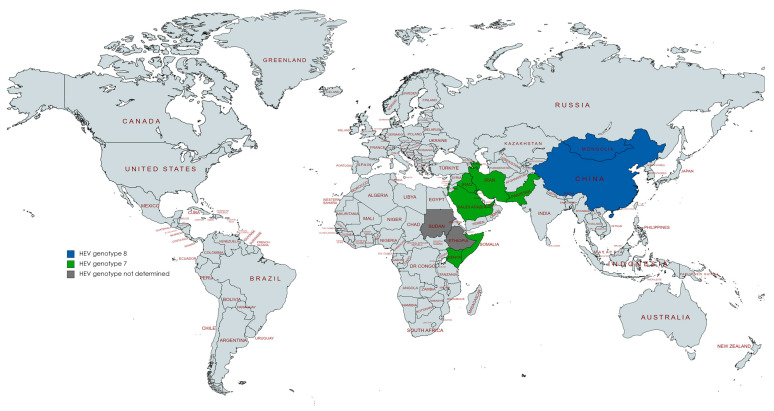
Countries with reports of HEV detection in camels.

All studies reporting HEV detection in camels were from the United Arab Emirates (*n* = two), Ethiopia (*n* = two), Israel (*n* = two), Mongolia (*n* = two), China (*n* = two), Saudi Arabia (*n* = one), Somalia (*n* = one), Sudan (*n* = one), Egypt (*n* = one), Kenya (*n* = one), or Pakistan (*n* = one), (Figure 2). Some papers studied samples from various countries. All these studies were focused on dromedary and Bactrian camels. Most of the studies were conducted with serum samples (*n* = six), four with stools, and one with milk. Detection of HEV in camels was mostly based on HEV RNA assays using RT-PCR (*n* = nine) and RT-nested PCR (*n* = one).

### 3.1. HEV in Camels

The first study of HEV in camel species was conducted in the UAE in fecal samples from adult dromedary, with HEV RNA being found in 1.48% (3/203) [23]. Authors reported for the first time a new HEV genotype, which was termed dromedary camel HEV (DcHEV), and later characterized as genotype seven [8]. The viral loads of the three DcHEV-positive samples were 3.7 × 10^5^, 4.5 × 10^5^, and 3.2 × 10^7^ copies/mL. The complete-genome sequence of two DcHEV strains revealed that the genome size was 7220 bp and had a G + C content of 55% [23]. Two years later, the same authors reported a new HEV genotype (genotype eight) in stools from farmed Bactrian camels in China [24]. In this study, HEV was found in 1.5% (3/205) of samples with viral loads of 1.6 × 10^3^, 2.1 × 10^3^, and 1.8 × 10^4^ copies/mg. Whole-genome sequencing of the three positive samples showed genome sizes of 7212–7223 bp and a G + C content of 52.7–53.1% [24].

Another study conducted on serum and stool samples of dromedary camels from several countries, namely the UAE, Somalia, Sudan, Egypt, Kenya, and Pakistan [25], also found evidence of HEV infection in this species. HEV RNA was detected in 0.6% (12/2171) of serum samples and in 1.9% (5/267) of fecal samples; all the isolates were typed as the new genotype seven. Viral loads ranged from 3.2 × 10^4^ to 3.6 × 10^7^ IU/g in stools and 6.2 × 10^2^ to 8.3 × 10^6^ IU/mL in serum.

Two studies conducted in East Asia also detected HEV in camels. The first study was carried out in China in stool samples from Chinese Bactrian camels [45], which detected HEV RNA in 1.4% (4/295) of samples, but no genotyping characterization was conducted. The other study was conducted in Mongolia and found HEV RNA in 1% (2/200) of serum samples of Bactrian farmed camels [7], being typed as genotype eight.

Moreover, two more studies were carried out in the Middle East, namely in Israel and Saudi Arabia. The study from Israel used serum samples from local dromedaries [26], with HEV RNA being detected in 0.7% (1/142) of samples and typed as genotype seven. The study from Saudi Arabia used serum samples from imported dromedary camels from Sudan and Djibouti [27]. Samples were collected from three camel farms and an abattoir located in Jeddah city before the camels were slaughtered [44]. The detection of HEV RNA showed a prevalence rate of 1.77% (21/1189), with a higher occurrence of DcHEV RNA found in domestic dromedaries (12/296,4.1%) in comparison with imported dromedaries (9/893, 1.0%). Molecular characterization was accomplished on 19 out of 21 sequences, with all typed as genotype seven.

In a study performed in Ethiopia on fecal samples from camels kept under farmers’ management conditions, HEV RNA was detected in 2.2% (1/45) of samples, but the molecular characterization was not performed [46].

### 3.2. Reports of No Evidence of HEV in Camels

During recent years, many studies have also attempted to detect HEV in dromedary and Bactrian camels, but without success. The aforementioned research was carried out in China, where milk samples obtained from Bactrian camels native to the country were analyzed (*n* =20) [45], in Ethiopia and Mongolia with dromedary and Bactrian camel serum samples (*n* = 98) [43], and in Israel with camel serum samples of Bedouin households (*n* = 86) [44].

### 3.3. Molecular Diversity of Camel HEV

**Figure 3:**
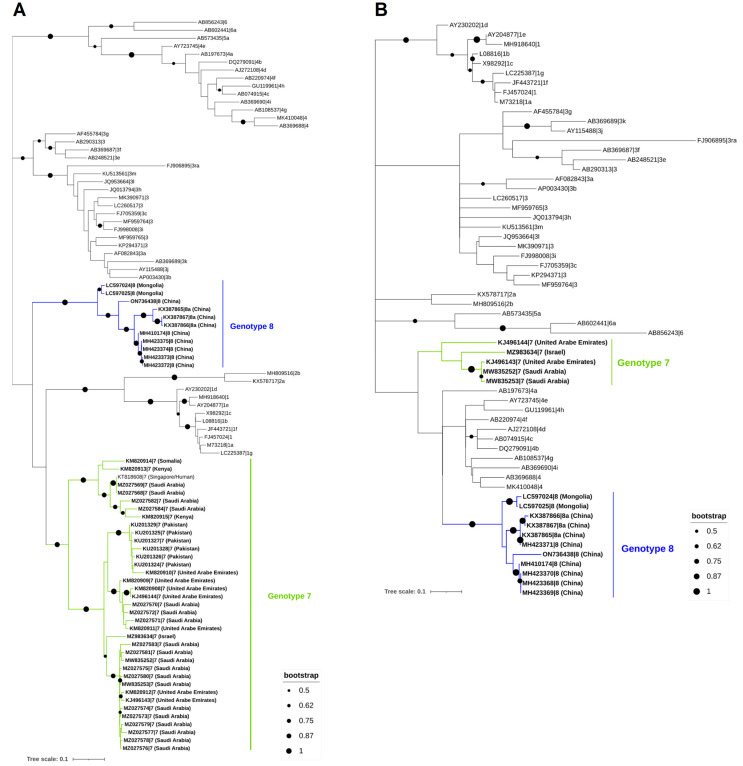
Phylogenetic analysis of HEV sequences found in dromedary and Bactrian camels. The 309 nt from the RNA-dependent RNA polymerase region in ORF1 (**A**) and 205 nt from the ORF2 (**B**). For both trees, ORF1 (**A**) and ORF2 (**B**), the maximum likelihood approach was used to build them (Kimura 2-parameter) in MEGA X and the Interactive Tree of Life (iTOL). The ORF1 tree (**A**) was created using 92 nucleotides of HEV sequences from the RNA-dependent RNA-polymerase region, including 47 camel HEV sequences available in GenBank as of 9 February 2023 (HEV-7 and -8, which are highlighted in bold), as well as 45 strains from other genotypes (HEV-1 to HEV-6, not highlighted or shaded, and identified by their accession number, genotype, and subgenotype). The ORF2 tree (**B**), on the other hand, was based on 60 nucleotides of HEV sequences from the ORF2 region, including 16 camel HEV sequences available in GenBank as of 9 February 2023 (HEV-7 and -8, highlighted in bold), as well as 44 strains from other genotypes (HEV-1 to HEV-6, not highlighted or shaded, and identified by their accession number, genotype, and subgenotype).

To establish the evolutionary connection of HEV in camels in a methodical manner, we collected all available sequences from the GenBank database. This compilation includes 11 complete genomes, 1 partial genomic sequence, 35 short partial sequences of the RNA-dependent RNA-polymerase (*RdRp*) gene, and 4 small partial sequences of the ORF2. Notably, the small partial HEV-7 sequences are located at the *RdRp* gene of the viral genome due to the use of an RT-PCR assay targeting the preserved HEV gene (Figure 3). The phylogenetic analysis of HEV sequences carried out in this review demonstrated that the dromedary camel HEV sequences clustered with the Bactrian camel HEV sequences, which are primarily found in Middle Eastern nations. The mean pairwise distances between each HEV genotype and between HEV genotypes seven and eight are described in Table 2.

## 4. Discussion

HEV is endemic in many Middle Eastern and African countries [47], and in Southeast Asia [2], where genotypes seven and eight have been detected in dromedary and Bactrian camels [7,23,24,25,26,27]. In general, the zoonotic nature of HEV was established through the identification and characterization of HEV (genotype three) isolated from swine, which showed to be genetically closely related to HEVs detected in humans [48]. Moreover, several reports of HEV transmission through the consumption of swine products or frequent contact with swine have led to the recognition of HEV as an important zoonotic agent [49]. In developed and industrialized countries, HEV infection appeared to be far more common than previously recognized [50] and should be considered a foodborne zoonosis, which can be acquired by the consumption of raw and undercooked products such as pork and dairy products [28]. For these reasons, HEV is considered a major public health problem worldwide and, therefore, studies on HEV in camels should be performed to clarify the infective potential of genotypes seven and eight in these animals.

Based on the georeferentiation of the samples, our results highlighted that HEV in camels was detected in regions where camel products are frequent parts of the human diet, with the most important camel products being milk and meat [34]. Therefore, HEV-contaminated products can enter the food chain and infect other animals and humans given the higher exposure to the virus in these territories [26]. In addition, the exposure can also be associated with the use of these animals for transporting materials, water, and other goods [34]. In contrast with other genotypes, such as HEV-3 and -4 in pork meat and pork products [51,52,53], there is still a lack of information about the transmission of HEV through the contact and consumption of camel products. In this sense, and since the transmission of HEV from swine to other animal species has been demonstrated [48,54], HEV transmission from camels to other animals, including humans, could also present the same risk.

The phylogenetic analysis of the camel HEV sequences performed in the present work showed that the HEV strains isolated from dromedary camels all fall into the same genotype (HEV-7), and the strains isolated from Bactrian camels fall into genotype eight. Furthermore, both dromedary and Bactrian camels’ HEV strains are included in the same clade. These strains are mostly present in Middle Eastern countries [7,23,24,25,26,27]. Remarkably, there is still a lack of HEV evidence in countries with a large number of camel populations, such as Niger, Chad, India, and Pakistan [29], and where these animals have an important impact on social and economic activities [34], thus reinforcing that further studies on HEV in camels from these regions should be performed in order to understand its social and public health impact. Regarding the pairwise distance analysis performed for ORF1 and ORF2 and taking into account the sequences assigned as reference for genotypes seven and eight [8], it is observed that most of the sequences for each genotype show greater similarity with one of the reference sequences. Specifically, for genotype seven, most sequences show the highest similarity to the reference strand KJ496143 for both ORF1 and ORF2 regions. On the other hand, for genotype eight, most sequences show the highest similarity to the reference strand MH410174 of ORF1, while the majority of ORF2 sequences share the most similarity to the reference strand KX387865. Based on this analysis, it may be suggested that most of the HEV genotype seven sequences circulating in the environment are close to the KJ496143 strain. On the other hand, differences between the two regions (ORF1 and ORF2) have been observed in genotype eight, which could explain the higher variability between strains.

To date, there are no studies evaluating the clinical signs in camels infected by HEV. Thus, there is no indication of the need for disease control measures. Nonetheless, camels excrete HEV through their feces into the environment, which can potentially transmit the virus to another host [26]. In fact, a case of infection with camel-associated HEV was reported where a person from the United Arab Emirates with chronic hepatitis, who had undergone a liver transplant, was infected with HEV genotype seven [28]. The patient had a habit of regularly consuming camel meat and milk, indicating that this strain of HEV can indeed infect humans. This also implies that the consumption of food products derived from camels could be linked to post-transplantation hepatitis E. While the prevalence of genotype seven in the human population is mostly unknown, it is very common in dromedaries [25], particularly in camel calves during the first year of life [55]. Moreover, circulation of HEV genotype seven has been demonstrated in other countries with a high density of camel populations, such as Ethiopia, where a seroprevalence of 22.4% was described in dromedary and Bactrian camels [43] and a 1.5% rate of active HEV infection was detected in the camel population [25]. On the other hand, a new genotype, classified as genotype eight, has been identified in Bactrian camels in China [24]. This genotype, unlike genotype seven, has not been detected in humans; however, its zoonotic potential has been demonstrated in an in vivo model [45]. The evaluation of camel genotypes (seven and eight) in human and animal populations is limited, mainly due to the lack of specificity of current molecular techniques for the detection of these genotypes. Therefore, their epidemiology and circulation in animal species other than camels is still unknown. Thus, their importance in public health remains to be clarified. As such, it is essential to develop molecular diagnostic tools that demonstrate high sensitivity for camel genotypes, as well as for potential novel emerging genotypes. As new HEV variants are constantly being discovered in animals worldwide, our understanding of HEV diversity consequently continues to evolve [56]. Experts suggest that a One Health approach is necessary to comprehend and prevent HEV transmission [26]. To mitigate the risks of acquiring HEV during animal husbandry and production, it is essential to understand the distribution of this zoonotic virus in its animal reservoir [55]. HEV has been detected in different animals, including mongoose, horse, rhesus monkey, hare, sheep, goat, donkey, cow, yak, tree shrew, bottlenose dolphin, and vulture [57]. However, it is still unclear whether these animals are real HEV reservoirs or simply infected as a result of spillover [10].

## 5. Conclusions

Considering camels as utility animals in several countries and given that HEV in camels may pose a potential public health risk, further studies are indicated to assess the global prevalence of HEV infection in camels and to evaluate the risk of foodborne transmission from contaminated camel products.

## Figures and Tables

**Table 1:** Concise description of the research papers that have reported the detection of the Hepatitis E Virus (HEV) in camels.

Reference	Sampling Location	Species/Population Details	Sample Type	HEV Diagnostic Assay	Target Region (Molecular Test)	Number of Positives/Total	HEV Genotype
[23]	United Arab Emirates (UAE)	Dromedary camels	Stool	RT-PCR	ORF2	3/203	7
[24]	China	Farmed Bactrian camels	Stool	RT-PCR	ORF2	3/205	8
[25]	UAE, Somalia, Sudan, Egypt, Kenya, Pakistan	Dromedary camels	Serum and stool	RT-PCR	*RdRp*	Serum: 12/2171 Stool: 5/267	7
[43]	Ethiopia, Mongolia	Dromedary and Bactrian camels	Serum	RT-PCR	ORF1	0/98	ND
[44]	Israel	Dromedary camels	Serum	RT-PCR		0/86	ND
[45]	China	Bactrian camels	Milk and stool	RT-PCR	ORF1/ ORF2	Milk: 0/20 Stool: 4/295	ND
[26]	Israel	Dromedary camels	Serum	RT-PCR		1/142	7
[7]	Mongolia	Bactrian camels	Serum	RT-PCR	ORF2/ORF3	2/200	8
[46]	Ethiopia	Bactrian camels	Stool	nRT-PCR	ORF1	1/45	ND
[27]	Saudi Arabia, Sudan, Djibouti	Dromedary camels	Serum	RT-PCR	*RdRp*	21/1189	7

RT-PCR—Reverse-Transcriptase Polymerase Chain Reaction; nRT-PCR—Nested Reverse-Transcriptase Polymerase Chain Reaction; ORF—Open Reading Frame; *RdRp*—RNA-Dependent RNA-Polymerase; ND—not determined.

**Table 2:** The analysis involved 96 nucleotide sequences and focused on the number of base substitutions per site between sequences, as well as the mean pairwise distances between each HEV genotype and between HEV genotypes seven and eight. The final dataset contained 308 positions in total.

	Pair Wise Distance (%)			
HEV Genotypes	ORF1		ORF2	
	7	8	7	8
1	54.17–72.76	49.38–65.57	78.29–83.61	75.22–83.75
2	52.43–65.96	51.90–63.54	78.29–81.28	75.22–81.37
3	51.40–73.43	57.72–74.89	74.00–83.03	72.04–83.16
4	41.03–69.70	49.81–66.88	76.46–86.44	77.07–86.65
5	49.00–62.93	56.17–63.24	80.10–82.45	75.22–80.10
6	47.89–67.43	51.78–62.52	75.22–78.29	72.68–78.93
7	64.11–100	51.22–75.74	83.90–88.29	80.69–85.88
8	51.21–75.74	79.44–100	78.90–85.90	89.27–100

## Data Availability

The data presented in this study are available on request from the corresponding author.
